# First Records of Two Invasive Weevils (Coleoptera, Curculionidae, Dryophthorinae) in Brazil with Quarantine Potential

**DOI:** 10.1007/s13744-026-01367-w

**Published:** 2026-02-20

**Authors:** Aline de Oliveira Lira, Andrezo Adenilton Santos, Helio Nunes Barbosa da Silva, Paschoal Coelho Grossi

**Affiliations:** Lab de Taxonomia de Insetos, Dept of Agronomy, Univ Federal Rural de Pernambuco, Recife, Pernambuco Brazil

**Keywords:** Alien species, Banana production, Coconut production, Introduced pest, Rhynchophorini

## Abstract

We report the first records in Brazil of *Diocalandra frumenti* (Fabricius) and *Polytus mellerborgii* (Boheman), weevil species of potential phytosanitary concern. These detections were made through surveys in the Entomological Collection of the Universidade Federal Rural de Pernambuco (CERPE) and targeted field inspections in northeast Brazil. These findings expand the known distribution of these weevils in Brazil and underscore the need for enhanced phytosanitary surveillance due to their potential impact on crop production in other countries and the implications for quarantine regulations.

## Introduction

Dryophthorinae, a subfamily of Curculionidae, includes about 1200 species in 153 genera and ten tribes (Anderson and Marvaldi 2014; Bouchard et al. 2024). Several species are serious agricultural pests of palms, bananas, rice, maize, sugarcane, and bromeliads (Anderson and Marvaldi 2014; Chamorro et al. 2021). Many of these pest species are widely distributed, particularly in tropical and subtropical regions (Chamorro et al. 2021). Due to their adaptability to diverse host plants and ability to establish in new environments, these weevils present a significant threat to global crop production and international trade. Their global spread is often facilitated by accidental introductions through international trade in ornamental and agricultural plants, combined with biological adaptability and increasing global connectivity (Chamorro et al. 2021; Skendžić et al. 2021; Hoddle et al. 2024).

Early detection of invasive weevils relies on a combination of strategies, including surveillance by phytosanitary agencies, incidental detection by local extension specialists, taxonomic identification by researchers, and community reporting (Hester and Cacho 2017; Blackburn et al. 2020; Epanchin-Niell et al. 2021). These detections highlight the need for proactive monitoring systems, rapid response protocols tailored to agricultural and peri-urban landscapes, and robust taxonomic frameworks for the fast and accurate identification of invasive species.

In this context, we report two new records of Dryophthorinae species, tribe Rhynchophorini, in Brazil, each with potential quarantine significance. These records were obtained through systematic surveys conducted in 2022 at the Entomological Collection of Universidade Federal Rural de Pernambuco (CERPE). In 2022, during curatorial work in the Curculionoidea section of CERPE, six specimens of Rhynchophorini were discovered, all of which were collected manually near a fragment of the Atlantic Forest in Pernambuco State, Recife mesoregion, northeast Brazil. After morphological identification and literature review, two specimens were identified as *Diocalandra frumenti* (Fabricius) and two as *Polytus mellerborgii* (Boheman). All identifications were confirmed by Dr. Lourdes Chamorro (USDA-ARS, Systematic Entomology Laboratory).

Given the known associations of *D. frumenti* with coconut palm (*Cocos nucifera* L., Arecaceae) and *P. mellerborgii* with banana (*Musa* L. spp., Musaceae), we conducted targeted inspections in banana and coconut areas on the UFRPE campus in Recife, a site used for educational purposes. Although our primary objective here is to report the occurrence of these species, in the following sections, we present additional information on their biology, geographic distribution, and morphological features.

## *Diocalandra frumenti* (Fabricius)—a palm-associated weevil with quarantine concern

**Fig. 1:**
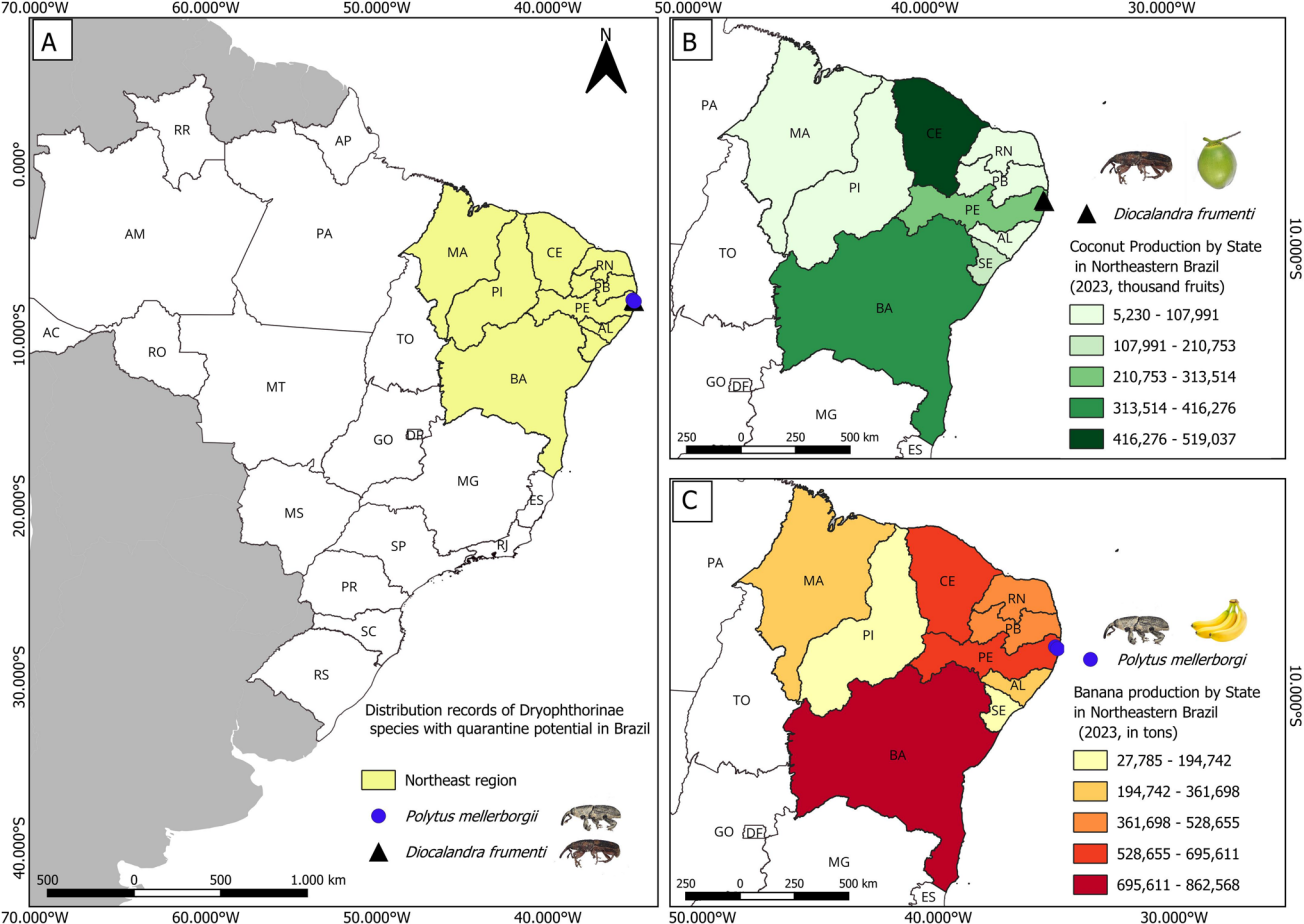
Distribution records for two Dryophthorinae species with quarantine relevance in Northeastern Brazil (**A**). Coconut production (in thousand fruits) by state in Northeastern Brazil in 2023, according to the IBGE (2025), and the locality where *Diocalandra frumenti* was recorded (**B**). Banana production (in tons) by state in Northeastern Brazil in 2023 (IBGE), and the locality where *Polytus mellerborgii* was recorded (**C**)

**Fig. 2:**
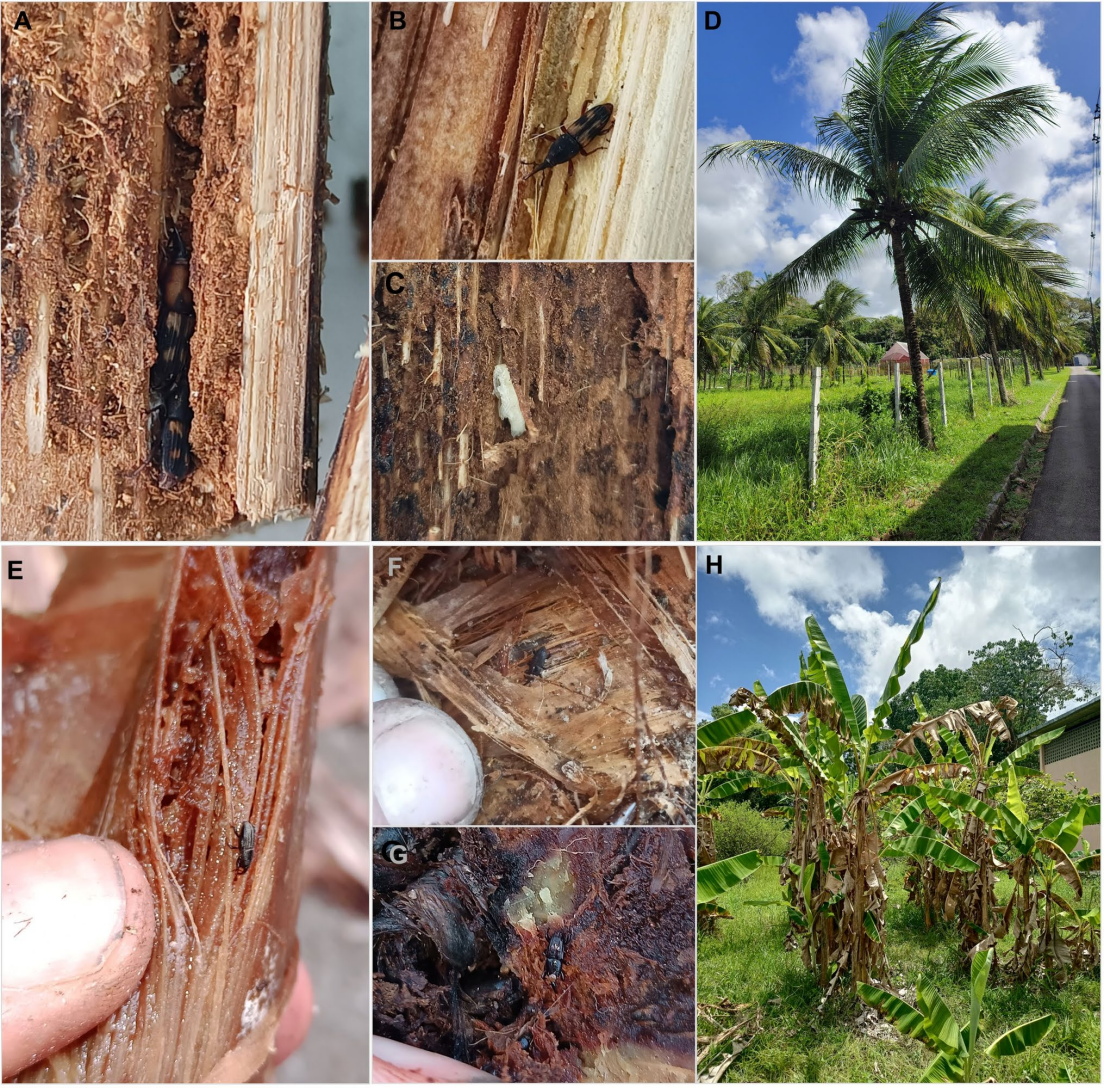
Injury signals of presence of *Diocalandra frumenti* and *Polytus mellerborgii* in coconut (**A**–**D**) and banana (**E**–**H**) plants. Adults (**A** and **B**) and pupa (**C**) of *Diocalandra frumenti* on petioles of coconut plants collected on Pernambuco (**D**). Adults of *Polytus mellerborgii* feeding on pseudostems (**E** and **F**) and rhizome (**I**) of banana plants in Pernambuco (**H**)

Four specimens of *D. frumenti* were discovered in CERPE in 2022, collected manually near Atlantic Forest fragments in Camaragibe, Pernambuco (Fig. 1A, B). Targeted inspections of coconut palms (*Cocos nucifera* L.) on the UFRPE campus, Recife, yielded 61 additional adult specimens from galleries in leaf sheaths and petioles (Fig. 2A–D).

Native to the coastal regions of the Indian Ocean, *D. frumenti* is widely distributed across Asia, Africa, Oceania, and parts of Europe and South America (Hill 1983; Núñez et al. 2002; Xu et al. 2012; Kojima et al. 2017; Vacas et al. 2017; Gil et al. 2018; Nguyen et al. 2020). In Brazil, it was previously listed as absent (MAPA 2025), but this record represents its first confirmed occurrence in Pernambuco. Additionally, an independent record of the genus was recently posted on the *iNaturalist* platform, documenting an individual in the municipality of Campos dos Goytacazes, state of Rio de Janeiro, in November 2024 (https://www.inaturalist.org/observations/253225277).

**Fig. 3:**
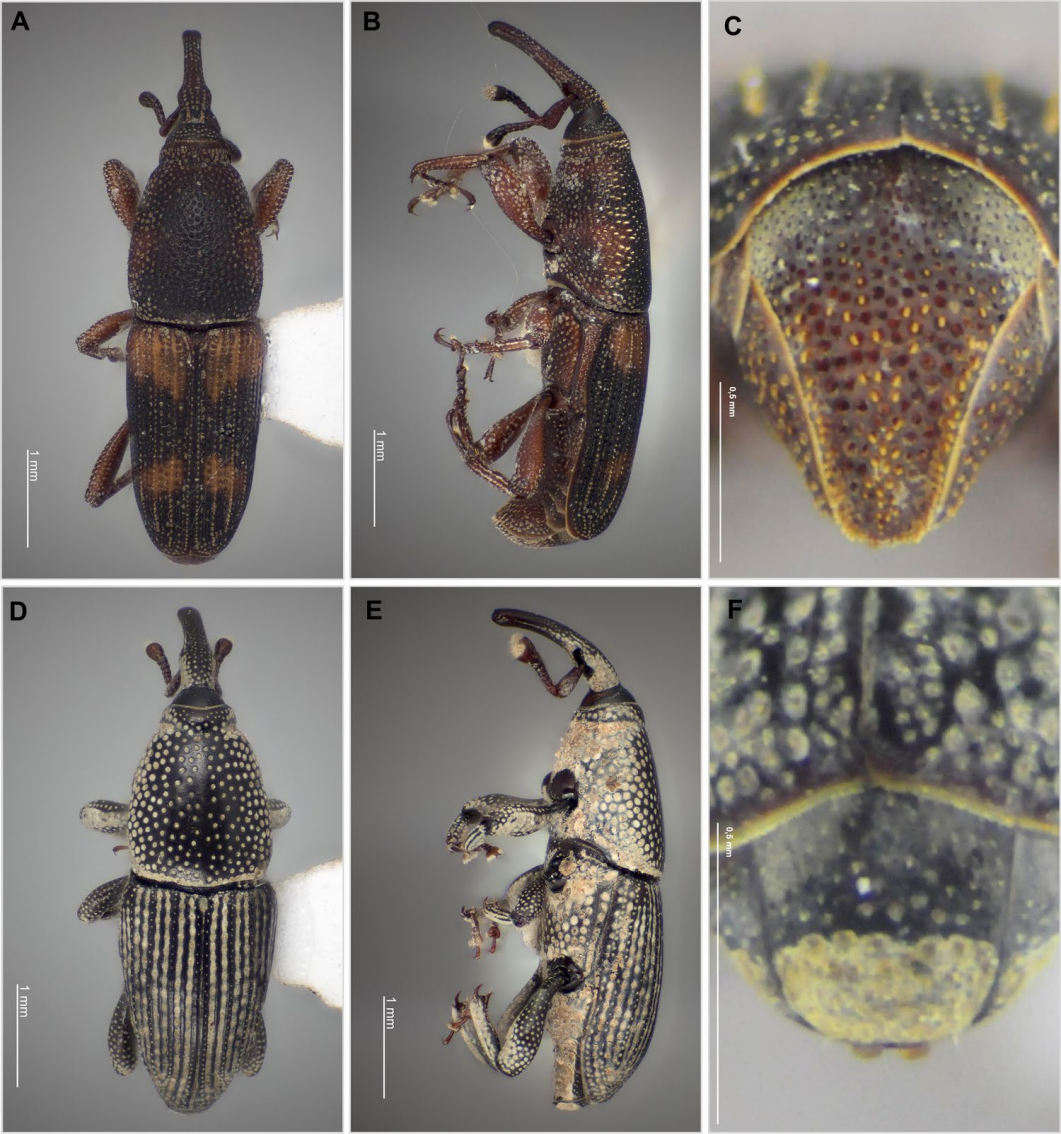
Dorsal view (**A**), lateral view (**B**), and pygidium (**C**) of *Diocalandra frumenti*. Dorsal view (**D**), lateral view (**E**), and pygidium (**F**) of *Polytus mellerborgii*

Adults of *D. frumenti* are shiny black, about 6–8 mm in length, with four red to brownish-yellow elytral spots (Fig. 3A, B). The pygidium lacks a sulcus and is covered with erect setae arranged in two to three median rows centrally and one lateral row on each side (Fig. 3C). Females oviposit in the crevices of palm stems, where they hatch within 4–9 days. Larvae develop over 8–10 weeks, feeding internally, and pupate 10–12 days before adult emergence (Howard et al. 2001). This species attacks at least 17 genera of Arecaceae, many of which are economically significant palms cultivated for food or landscaping, such as *C. nucifera*, *Phoenix dactylifera* L., *Phoenix canariensis* Hort. ex Chabaud, and *Elaeis guinensis* Jacq (Vacas et al. 2017). The larvae of *D. frumenti* primary damage in the basal third of the leaf rachis by boring into the tissues, creating galleries that cause exudation, leading to premature desiccation and collapse (Salomone-Suárez et al. 2000; Ramos-Cordero et al. 2024). However, they can also bore into roots, petioles, inflorescences, fronds, leaf sheaths, and fruits, damaging the trunk at various heights. This damage weakens plants and promotes microbial infections (Singh and Barrikkad 2017; Vacas et al. 2017). Larvae create 1–2-mm galleries in the rachis basal third, disrupting vascular bundles. Severe attacks may kill trees within 6–8 months (Gil et al. 2018; Núñez et al. 2002; Ramos-Cordero et al. 2024).

*Diocalandra frumenti* is a key pest of coconut crops worldwide (Singh and Barrikkad 2017; Nguyen et al. 2020). In 2023, Pernambuco, the first Brazilian state reporting the presence of this species, produced 245.8 million fruits on 7790 hectares (IBGE 2025). Given Brazil is the fifth-largest coconut producer, with production concentrated mainly in coastal areas of the Northeast and North (IBGE 2025) (Fig. 1B), the establishment of *D. frumenti* may have serious economic implications.

## *Polytus mellerborgii* (Boheman)—a cryptic banana pest in Northeastern Brazil

Two specimens were found in CERPE in 2022, collected in Pernambuco. Subsequent surveys in banana plantations on the UFRPE campus recovered dozens of adults from rhizomes and pseudostems by a manual sampling (Fig. 2E–H). In addition to this first confirmed record in Brazil, a recent observation in an urban area in Rio de Janeiro, Brazil, posted on iNaturalist (https://www.inaturalist.org/observations/71558650) also suggests a wider distribution.

Native to Southeast Asia, *P. mellerborgii* has been recorded in several regions across Asia, Europe, and the Americas, including Ecuador, Mexico, and more recently Colombia (Zimmerman 1941, 1968; González et al. 2007; Muñoz-Ruiz 2007; Núñez et al. 2002; Sepúlveda-Cano and Rubio-Gómez 2009; Peck 2017; de la Pava et al. 2020; MNHN and OFB 2025).

*Polytus mellerborgii*, commonly known as the small banana weevil (in Portuguese, “broca-pequena-da-bananeira”), is morphologically most similar to its congener *Cosmopolites sordidus* (Germar), another banana weevil borer. This species measures approximately 4 mm in length, has a black coloration, and exhibits dense, deep punctures covering most of the body (Fig. 3D–F). Males possess a large pygidial plate located near ventrites III to V, strongly projected downward, while females have a smaller pygidial plate projected forward (Sepúlveda-Cano and Rubio-Gómez 2009). It is primarily nocturnal and completes its life cycle inside banana rhizomes and pseudostems, typically in decaying tissues (Fig. 2E–G) (Ramírez and Espinosa 2009). Eggs are laid in wounds or decaying tissues, and larvae feed internally, creating galleries and potentially weakening the plant (González et al. 2007; Orellana 2007; Ramírez and Espinosa 2009; Yin et al. 2016).

Bananas are among the most widely consumed fruits globally (Medina and Ruales 2024; Garcés-Moncayo et al. 2025) and a major crop in Brazil, which ranks fourth in global production (IBGE 2024). Despite the socioeconomic importance of the banana agribusiness in Brazil, phytosanitary issues remain a significant challenge for the crop. The recent detection of *P. mellerborgii* in Pernambuco, the fifth-largest banana-producing state in Brazil (IBGE 2024) (Fig. 1C), may represent an additional limiting factor that warrants the attention of regulatory agencies and producers. Notably, the last record of the introduction of this species in South America was reported recently in Colombia (de la Pava et al. 2020), highlighting its rapid dispersion across major banana-growing regions on the continent. Although the impacts of *P. mellerborgii* in Brazil are still uncertain, its presence could represent a new challenge for the national banana industry.

## Concluding remarks

These new records expand the known distribution of *D. frumenti* and *P. mellerborgii*, highlighting their potential quarantine relevance. The detections underscore the vulnerability of Brazil to the invasive Dryophthorinae, especially given intense global trade and climatic suitability, which can facilitate the rapid movement and establishment of pests. The limited knowledge of the biology and ecology of these species in the Brazilian context reinforces the need for further research to evaluate their potential impacts on agriculture and native ecosystems. Investigations into their life cycles, host preferences, dispersal mechanisms, and interactions with native species are essential to assess their status as pests and guide appropriate management strategies. Accurate taxonomic identification remains necessary for rapid response and effective phytosanitary action. These findings reinforce the need for updated pest risk assessments, continuous monitoring, and improved regulation of plant material movement.

## References

[CR1] Anderson RS, Marvaldi A (2014) Dryophthorinae Schoenherr, 1825. In: Kristensen NP, Beutel RG, Leschen RAB (eds) Handbook of zoology. Arthropoda: Insecta. Volume 3. Coleoptera, beetles. Morphology and systematics. Berlin, Boston, pp 477–483

[CR2] Blackburn GS, Bilodeau P, Cooke T, Cui M, Cusson M, Hamelin RC, Keena MA, Picq S, Roe AD, Shi J, Wu Y (2020) An applied empirical framework for invasion science: confronting biological invasion through collaborative research aimed at tool production. Ann Entomol Soc Am 113(4):230–245. 10.1093/aesa/saz072

[CR3] Bouchard P, Bousquet Y, Davies AE, Cai C (2024) On the nomenclatural status of type genera in Coleoptera (Insecta). ZooKeys 1194:1–981. 10.3897/zookeys.1194.106440 38523865 10.3897/zookeys.1194.106440 PMC10955229

[CR4] Chamorro ML, de Meiros BAS, Farrell BD (2021) First phylogenetic analysis of Dryophthorinae (Coleoptera, Curculionidae) based on structural alignment of ribosomal DNA reveals Cenozoic diversification. Ecol Evol 11(5):1984–1998. 10.1002/ece3.7131 33717436 10.1002/ece3.7131 PMC7920784

[CR5] IBGE Instituto Brasileiro de Geografia e Estatística (2024) Tabela 7832: Área plantada, área colhida, quantidade produzida, rendimento médio e valor da produção da lavoura permanente. Sistema IBGE de Recuperação Automática – SIDRA. https://sidra.ibge.gov.br/tabela/7832. Accessed 19 Jun 2025

[CR6] IBGE Instituto Brasileiro de Geografia e Estatística (2025) Coco-da-baía. Produção Agropecuária. https://www.ibge.gov.br/explica/producao-agropecuaria/coco-da-baia/br. Accessed 19 Jun 2025

[CR7] de la Pava N, García MA, Brochero CE, Sepúlveda-Cano PA (2020) Registros de Dryophthorinae (Coleoptera: Curculionidae) de la Costa Caribe colombiana. Acta Biol Colomb 25(1):96–103

[CR8] Epanchin-Niell R, Thompson AL, Treakle T (2021) Public contributions to early detection of new invasive pests. Conserv Sci Pract 3(6):e422. 10.1111/csp2.422

[CR9] Garcés-Moncayo MF, Guevara-Viejó F, Valenzuela-Cobos JD, Galindo-Villardón P, Vicente-Galindo P (2025) Modeling of the physicochemical and nutritional composition of *Musa paradisiaca* (Williams Variety) at different ripening stages in Ecuador. Agriculture 15(10):1025. 10.3390/agriculture15101025

[CR10] Gil JRE, Suárez EH, Barrallo ES (2018) Estudio de la metodología de cría de Diocalandra frumenti (Fabricius, 1801) (Coleoptera: Dryophthoridae). Granja Revista Agropecuaria 23:108–117

[CR11] González C, Aristizábal M, Aristizábal J (2007) Dinámica poblacional de picudos en plátano (*Musa* AAB) Dominico hartón. Agron 15(2):33–38

[CR12] Hester SM, Cacho OJ (2017) The contribution of passive surveillance to invasive species management. Biol Invasions 19:737–748

[CR13] Hill DS (1983) Agricultural insect pests of the tropics and their control. Cambridge University Press, Cambridge, England

[CR14] Hoddle MS, Antony B, El-Shafie HA, Chamorro ML, Milosavljević I, Löhr B, Faleiro JR (2024) Taxonomy, biology, symbionts, omics, and management of *Rhynchophorus* palm weevils (Coleoptera: Curculionidae: Dryophthorinae). Annu Rev Entomol 69(1):455–479 38270987 10.1146/annurev-ento-013023-121139

[CR15] Howard FW, Giblin-Davis R, Moore D, Abad R (2001) Insects on palms. Cabi, New York

[CR16] Kojima H, Kidokoro H, Tsuru T (2017) First occurrence of the lesser coconut weevil, *Diocalandra frumenti* (Coleoptera, Dryophthoridae) in the Ogasawara Islands, Japan. Elytra 7:239–240

[CR17] Medina MDR, Ruales J (2024) Post-harvest alternatives in Banana Cultivation. Agronomy 14:2109. 10.3390/agronomy14092109

[CR18] MAPA Ministério da Agricultura, Pecuária e Abastecimento (2025) Portaria SDA/MAPA Nº 1.291, de 22 de Maio de 2025. Atualiza a lista de pragas quarentenárias ausentes (PQA) para o Brasil. Diário Oficial da República Federativa do Brasil, Brasília, 27 Maio de 2025. Seção 1, pp 9**–**13. https://www.gov.br/agricultura/pt-br/assuntos/sanidade-animal-e-vegetal/sanidade-vegetal/analise-de-riscos-de-pragas. Accessed 8 Oct 2025

[CR19] MNHN and OFB (2003–2025) Sheet of *Polytus mellerborgii* (Boheman, 1838). Inventaire national du patrimoine naturel (INPN). https://inpn.mnhn.fr/espece/cd_nom/714828. Accessed 17 Jun 2025

[CR20] Muñoz-Ruiz C (2007) Fluctuación poblacional del picudo negro (*Cosmopolites sordidus* Germar) del plátano (Musa AAB) en San Carlos, Costa Rica. Rev Tecnol Marcha 20(1):24–41

[CR21] Nguyen HU, Nguyen TH, Chau NQK, Le VV, Tran VH (2020) Biology, morphology and damage of the lesser coconut weevil, *Diocalandra frumenti* (Coleoptera: Curculionidae) in southern Vietnam. Biodiversitas 21:4686–4694

[CR22] Núñez MG, Álvarez AJ, Salomone F, Carnero A, Del Estal P, Durán J (2002) *Diocalandra frumenti* (Fabricius) (Coleoptera: Curculionidae), nueva plaga de palmeras introducida en Gran Canaria. Primeros estudios de su biología y cría en laboratorio. Bol San Veg Plagas 28(3):347–355

[CR23] Orellana CA (2007) Descripción de las plagas del cultivo del banano de 1995 al 2002 en las fincas de Cobigua en el distrito de Entre Rios, municipio de Puerto Barrios, Izabal. Dissertation, Universidad de San Carlos de Guatemala

[CR24] Peck SB (2017) CDF Checklist of Galapagos beetles-FCD Lista de especies de Escarabajos Galápagos. In: Bungartz F, Herrera H, Jaramillo P, Tirado N, Jiménez-Uzcátegui G, Ruiz D, Guézou A, Ziemmeck F (eds) Charles Darwin Foundation Galapagos Species Checklist - Lista de Especies de Galápagos de la Fundación Charles Darwin. Charles Darwin Foundation / Fundación Charles Darwin, Puerto Ayora, Galapagos, pp 1-15

[CR25] Ramírez CM, Espinosa LFV (2009) Métodos de muestreo para evaluar poblaciones de picudos del plátano (Coleoptera: Curculionidae, Dryophthorinae) en el departamento de Caldas-Colombia. http://camilomedina.files.wordpress.com/2010/03/metodos-de-muestreo-para-picudos-del-platano2.pdf. Accessed 20 Jun 2025

[CR26] Ramos-Cordero C, Seris-Barrallo E, Vacas S, Navarro-Llopis V, Hernández-Suárez EM (2024) Effect of commercial trap design and location on captures of *Diocalandra frumenti* (Fabricius) (Coleoptera: Dryophthoridae) on Palm Trees. InSects 15(10):738 39452314 10.3390/insects15100738 PMC11508261

[CR27] Salomone-Suárez F, Carnero Hernández A, González Hernández A, Marrero Ferrer M (2000) Presencia en la zona palearctica de *Diocalandra frumentii* Fabricius, (Coleoptera, Curculionidae). Bol Asoc Esp Entomol 24(1–2):263–264

[CR28] Sepúlveda-Cano PA, Rubio-Gómez JD (2009) Especies de Dryophthorinae (coleoptera: curculionidae) asociadas a plátano y banano (*Musa* spp.) en Colombia. Acta Biol Colomb 14(2):49–72

[CR29] Singh AK, Barrikkad R (2017) Taxonomic redescription of the coconut bark weevil (*Diocalandra frumenti*). J Pharmacogn Phytochem 1:1049–1053

[CR30] Skendžić S, Zovko M, Živković IP, Lešić V, Lemić D (2021) The impact of climate change on agricultural insect pests. InSects 12(5):440. 10.3390/insects12050440 34066138 10.3390/insects12050440 PMC8150874

[CR31] Vacas S, Navarro I, Seris E, Ramos C, Hernández E, Navarro-Llopis V, Primo J (2017) Identification of the male-produced aggregation pheromone of the four-spotted coconut weevil, *Diocalandra frumenti*. J Agric Food Chem 65(2):270–275. 10.1021/acs.jafc.6b04829 27983833 10.1021/acs.jafc.6b04829

[CR32] Xu H, Qiang S, Genovesi P, Ding H, Wu J, Meng L, Han Z, Miao J, Hu B, Guo J, Sun H (2012) An inventory of invasive alien species in China. NeoBiota 15:1–26. 10.3897/neobiota.15.3575

[CR33] Yin J, Wang Y, Lu F, Gao J, Zhao D (2016) Antennal sensilla in the small banana weevil *Polytus mellerborgi* Boheman (Coleoptera: Curculionidae). Pak J Zool 48(2):527–531

[CR34] Zimmerman EC (1941) The Rhynchophorinae found in Hawaii (Coleoptera: Curculionidae). Proc Hawaii Entomol Soc 11:96–98

[CR35] Zimmerman EC (1968) Rhynchophorinae of Southeastern Polynesia (Coleoptera: Curculionidae). Pac inSects 10(1):47–77

